# Humoral and cellular immunity to SARS-COV-2 after vaccination with mRNA vaccines in PLWH with discordant immune response. Influence of the vaccine administered

**DOI:** 10.3389/fimmu.2023.1129753

**Published:** 2023-03-15

**Authors:** Luis F. López-Cortés, Abraham Saborido-Alconchel, María Trujillo-Rodríguez, Ana Serna-Gallego, Silvia Llaves-Flores, Esperanza Muñoz-Muela, María Jesús Pérez-Santos, Carmen Lozano, Marta Mejias-Trueba, Cristina Roca, Nuria Espinosa, Alicia Gutiérrez-Valencia

**Affiliations:** ^1^ Infectious Diseases and Clinic Microbiology Unit. Biomedicine Institute of Seville/Virgen del Rocío University Hospital/Consejo Superior de Investigaciones Científicas/University of Seville, Seville, Spain; ^2^ Pharmacy Service, Virgen del Rocío University Hospital, Seville, ;Spain

**Keywords:** people living with HIV, mRNA-1273 vaccine, BNT162b2 vaccine, anti-S RBD IgG, neutralizing antibodies, SARS-CoV-2 specific B cells, SARS-CoV-2 T cell response, SARS-CoV-2 vaccines

## Abstract

**Background:**

Data on SARS-CoV-2 mRNA vaccine immunogenicity in people living with human immunodeficiency virus (PLWH) and discordant immune response (DIR) are currently limited. Therefore, we compare the immunogenicity of these vaccines in DIR and immunological responders (IR).

**Methods:**

A prospective cohort that enrolled 89 participants. Finally, 22 IR and 24 DIR were analyzed before vaccination (T_0_), one (T_1_) and six months (T_2_) after receiving BNT162b2 or mRNA-1273 vaccine. Additionally, 10 IR and 16 DIR were evaluated after a third dose (T_3_). Anti-S-RBD IgG, neutralizing antibodies (nAb), neutralization activity, and specific memory B cells were quantified. Furthermore, specific CD4^+^ and CD8^+^ responses were determined by intracellular cytokine staining and polyfunctionality indexes (Pindex).

**Results:**

At T_1_, all participants developed anti-S-RBD. 100% IR developed nAb compared to 83.3% DIR. Spike-specific B cells were detected in all IR and 21/24 DIR. Memory CD4^+^ T cells responded in 5/9 IR and 7/9 DIR, mainly based on the expression of IFN-γ and TNF-α, with a higher Pindex in DIR. Memory CD8^+^ T cells responded in only four participants in each group. At T_2_, anti-S-RBD and nAb titers were higher in DIR than in IR. In both groups, there was an increase in specific B memory cells, higher in DIR. Six IR and five DIR maintained a specific memory CD4^+^ response. Memory CD8^+^ response was preserved in IR but was lost in DIR. In a multivariate linear regression analysis, receiving mRNA-1273 instead of BNT162b2 played a prominent role in the results.

**Conclusions:**

Our data suggest that PLWH with DIR can mount an immune response similar to those with higher CD4^+^, provided they receive the mRNA-1273 vaccine instead of others less immunogenic.

## Introduction

Since the emergence of the SARS-CoV-2 pandemic, vaccination has been the most effective strategy to decrease transmission, morbidity, and mortality. At least 26 vaccines have been evaluated in phase III clinical trials, according to WHO (1). Among them, the messenger RNA (mRNA) vaccines, mRNA-1273 and BNT162b2, achieve higher levels of antibodies than the viral DNA-vectored or inactivated COVID-19 vaccines, widely used with high effectiveness against COVID-19- infection, -related hospitalization and death (2–6).

Many studies indicate that the neutralizing SARS-CoV-2 antibodies elicited by these vaccines persist for more than six months; however, the antibody concentrations and vaccine effectiveness decrease over time. This setback and the emergence of new variants had made additional booster doses advisable (7–9).

On the other hand, the response to COVID-19 vaccines is lower among immunosuppressed subjects, including people living with human immunodeficiency virus (PLWH) (10). Several observational studies have been carried out in this population, mainly limited to serological analyses, with a short follow-up and a low representation of subjects with <200 CD4^+^/µl (11–25).

Among PLWH with low CD4^+^ T cell counts, there is a group that deserves special consideration, characterized by poor CD4^+^ T cell recovery despite sustained successful viral suppression, known as discordant immune responders (DIR). They show higher rates of morbidity and mortality associated with AIDS and non-AIDS events, in whom lower effectiveness of vaccines can be expected (26–29), and whose response to these vaccines has not been extensively characterized (30). Therefore, it would be advisable to characterize the immune response and the correlates of vaccine efficacy in these subjects. Furthermore, finding a predictive marker of response to vaccination would help schedule vaccinations to achieve an optimal response.

## Materials and methods

### Study participants

We conducted a prospective study of humoral and cellular immune responses against the original SARS-CoV-2 strain among PLWH without serological evidence of previous infection at the Virgen del Rocío University Hospital in Seville, Spain. Participants were selected based on CD4^+^ T cell recovery on antiretroviral treatment (ART) and classified as discordant immune responders (DIR), i.e., started ART with <350 CD4^+^ T cell/µl, maintained an undetectable viral load and had an increase of <200 CD4^+^ after 18 months of follow-up, and immunological responders (IR) who, under the same conditions, had an increase of CD4^+^ >350/µl.

Exclusion criteria included a diagnosis of SARS-CoV-2 infection, defined by a positive result of RT-PCR on a nasopharyngeal swab, anti-N or anti-S positive antibodies before the first vaccine dose (T_0_) or anti-N one month (T_1_) or six months (T_2_) after the second dose_-_ Likewise, pregnant women, neoplastic or autoimmune disease, treatment with steroids, immunomodulatory agents, and chemotherapy were excluded.

### Ethics statement

The study was carried out according to the principles of Good Clinical Practice after being approved by the Ethics Committee for Clinical Research of the Virgen Macarena and Virgen del Rocío University Hospitals (CEI VM-VR_04/2021_N) and the National Health Authority. The study is registered at ClinicalTrials.gov (NCT05633927). All participants provided signed informed consent.

### Endpoint, follow-up, and assessments

This study aimed to carry out a comprehensive longitudinal analysis of the three branches of adaptive immunity after two doses of the SARS-CoV-2 mRNA vaccines and up to 1 month after the third dose in a subset of PLWH, with particular attention to DIR on stable ART and undetectable viral load. The primary endpoints were to compare the presence of anti-SARS-CoV-2 spike RBD (total IgG) and surrogate neutralizing antibody (nAb) kinetics and specific memory B, CD4^+^, and CD8^+^ T cells for wild-type SARS-CoV-2 at T_1_ and T_2_ after two shots of SARS-CoV-2 mRNA vaccines in DIR and IR PLWH. Secondary endpoints were to assess whether these immune responses are associated with the phenotypic characteristics of peripheral blood T cell or plasma interleukin (IL) 4 and 21 concentrations in both groups of participants.

All study participants received two doses of the Pfizer–BioNTech BNT162b2 vaccine or the Moderna mRNA-1273 vaccine with a 21- or 28-day interval, respectively, between April and June 2021, and a third dose a median of 204 days (IQR, 175–212) later during the Spanish vaccination campaign. Blood samples were collected at the time of the first dose (baseline, T_0_), one (T_1_) and six months (T_2_) after the second dose, and one month after the third dose (T_3_).

Demographic and clinical variables related to HIV infection and the SARS-CoV-2 vaccination schedule were obtained anonymously for each subject through medical records.

### Cell and plasma isolation

Venous blood samples were collected in BD Vacutainer^®^ CPT™ mononuclear cell preparation tubes (with sodium heparin, BD Biosciences), and peripheral blood mononuclear cells (PBMC) were isolated by density gradient centrifugation on the same day of blood collection. Afterward, the PBMC were cryopreserved in a freezing medium (90% fetal bovine serum (FBS) (Gibco) + 10% dimethyl sulfoxide (DMSO) (PanReac AppliChem) in liquid nitrogen until further use.

Plasma samples were obtained using BD Vacutainer™ PET ethylenediamine tetraacetic acid (EDTA) centrifugation tubes and cryopreserved at -80°C until further use.

### Quantification of anti-N, anti-S RBD IgG, and surrogate-neutralizing antibodies

Anti-SARS-CoV-2 nucleocapsid and anti-SARS-CoV-2 spike RBD IgG antibodies were measured by ElectroChemiLuminescent Immuno Assays using the Elecsys Anti-SARS-CoV-2 assay and the Elecsys Anti-SARS-CoV-2 S assay (Roche Diagnostics. International Ltd, Switzerland), respectively, on a Cobas e 602 module analyzer. For the first one, the results were either reactive or nonreactive, as well as in the form of a cutoff index (COI; signal sample/cutoff). Therefore, COI ≥1.0 result was considered positive. For the second, the results were expressed in IU/ml (= BAU/ml).

Neutralizing antibody (nAb) concentrations against the receptor binding domain (RBD) of the SARS-CoV 2 spike protein S1 subunit were determined by a surrogate neutralization assay (SARS-CoV-2 NeutraLISA assay; Euroimmun AG, Lübeck, Germany) according to the manufacturer’s instructions.

The percentage of inhibition (% IH) was calculated as follows: 100% - [(average OD sample x 100%)/average OD blank]. Values were considered negative when % IH < 20, doubtful when % IH was between ≥ 20 to < 35, and positive when % IH reached ≥ 35%. The results were also expressed in IU/ml; the conversion between IU/ml and the neutralizing activity was performed according to the manufacturer’s instructions.

### Quantification of interleukin 4 (IL-4) and interleukin 21 (IL-21)

Determinations of IL-4 and IL-21 were performed using a multiplex bead-based immunoassay (MILLIPLEX^®^ MAP human high-sensitivity T cell magnetic bead panel; Merck EMD Millipore, Billerica, MA) following the manufacturer’s instructions. The assay was performed on the Luminex^®^ MAGPIX^®^ System detection instrument operated with xPONENT Software V4.2 (both from Luminex Corp., Austin, TX). The results were analyzed using the Belysa™ Analysis software V1.2.0 (Merck KGaA, Darmstadt, Germany).

### Immunophenotyping of T

For T cell phenotyping, the PBMC were stained with LIVE/DEAD Fixable Aqua Dead Cell Stain (Life Technologies), anti-CD4-FITC (clone RPA-T4), anti-CD8- PerCPCy5.5 (clone SK1), anti-CD3-BV711 (clone SP34-2), anti-CCR6-BV605 (CD196 clone 11A9), anti-PD-1-BV786 (CD279, clone EH12-1), anti-CD38-BV650 (clone HIT2), anti-CD25-Bv421 (clone M-A251), anti-CD27-APCH7 (clone M-T271), anti-CXCR3-APC (CD183 clone 1C6/CXCR3), anti-HLADR-AF700 (clone G46-6), anti-CD45RA-PE-Cy7 (clone L48) (all of them from BD Bioscience), and anti-CXCR5-PE/Dazzle 594 (clone J252D4) (from BioLegend). Afterward, the PBMC were washed with PBS, fixed and permeabilized with BD Cytofix/CytoPerm (BD Biosciences) according to the manufacturer’s instructions, and intracellularly stained at 4°C 30 min with anti-FoxP3-PE. Finally, the PBMC were fixed with a solution of 4% paraformaldehyde. T cells were gated according to CD3, CD4, and CD8 expression. T cell subsets (total memory, memory; central memory, CM; effector memory, EM; and terminally differentiated effector memory, TEMRA) were gated based on CD45RA and CD27 expression. In addition, T follicular helper cells (Tfh) were gated on CD4, CXCR5, CXCR3, and CCR6 expression, and Treg cells on CD4, CD25, and Foxp3 expression. The gating strategy is shown in **Supplementary Figure 1**.

### Detection of SARS-CoV-2-specific B cells

For the detection of SARS-CoV-2-specific B cells, the PBMC were stained with LIVE/DEAD Fixable Aqua Dead Cell Stain (Life Technologies), anti-CD14-BV510 (clone M5E2), anti-CD3-BV510 (clone UCHT1), anti-CD56-B510 (clone NMCAM16.2), anti-CD38-BV605 (clone HB7), anti-IgM-BV650 (clone G20-127), anti-IgG-BV421 (clone G18-145), anti-CD19-APC (clone SJ25C1), anti-IgD-AF700 (clone IA6-2), anti-CD20-PeCy7 (clone L27), anti-CD27-Pe (clone L128) (all of them from BD Bioscience) and anti-SARS-Cov-2 S protein-AF488 (clone P05DHu) (Abcam). In addition, the SARS-CoV-2 protein conjugated to AF488 was used to detect SARS-CoV-2 specific B cells. Briefly, the SARS-CoV-2 S1/S2-protein (also referred to as S-protein) (Sino Biological Inc.) was coupled with Alexa Flour 488 fluorochrome using the Lightning-Link^®^ Rapid Conjugation System (Abcam) according to the manufacturer’s protocol. B cells were gated according to CD3, CD14, CD56, and CD19 expression. B cell subsets [(naïve, memory, and antibody-secreting cells (ASC)] were gated based on IgD, CD27, and CD38 expression. The samples were acquired on a BD LSR Fortessa™ Cell Analyzer flow cytometer using FACS Diva software (BD Biosciences) and analyzed using FlowJo 10.7.1 software (Treestar, Ashland, OR). **Supplementary Figure 2** shows the gating strategy.

### Specific SARS-CoV-2 T cell responses

The PBMC were thawed, washed with RPMI 1640 (Gibco), and rested for 1 h in 0.25 µL/ml DNase I (Roche Diagnostics) containing complete R-10 medium [RPMI 1640 supplemented with 10% FBS, 100 U/ml penicillin G, 100 µg/ml streptomycin sulfate (Gibco) and 1.7 mM sodium L-glutamine (Lonza)]. One and a half million PBMCs were stimulated for 6 h at 37°C in R-10 medium with overlapping Spike protein peptides (PepMix™ SARS-CoV-2; Spike Glycoprotein, from JPT), 5 µg/ml of brefeldin A (Sigma Chemical Co), 2.6 µg/ml of monensin (Golgi Stop, BD Biosciences) protein transport inhibitors, anti-CD107a-BV650 (clone H4A3; BD Biosciences) monoclonal antibody and purified CD28 (clone CD28.2) and CD49d (clone 9F10) (BD Biosciences). Furthermore, 1.5 × 10^6^ PBMC without Spike protein peptides were included as a negative control.

Subsequently, the PBMCs were stained with a viability marker (LIVE/DEAD Fixable Aqua or Violet Dead Cell Stain; Life Technologies), anti-CD14-BV510 (clone MφP9), anti-CD19-BV510 (clone SJ25C1), anti-CD56-BV510 (clone NMCAM16.2), anti-CD8-APC (clone SK-1), anti-CD3-BV711 (clone SP34-2), anti-CD45RA-FITC (clone L48), anti-CD27-APCH7 (clone M-T271), anti-PD-1-BV786 (CD279, clone EH12-1) (BD Bioscience) and anti-TIGIT-PerCPCy5.5 (clone A15153G), anti-TIM3-PeCF594 (CD366, clone F38-2E2) and anti-LAG3-BV605 (CD223, clone 11C3C65) (BioLegend). The PBMC were washed with PBS and fixed and permeabilized with BD Cytofix/CytoPerm (BD Biosciences) according to the manufacturer’s instructions. Intracellular cytokine staining (ICS) was carried out at 4°C for 30 min with anti-IL-2-BV421 (clone MQ1-17H12), anti-IFN-γ-PE-Cy7 (clone B27) (BD Bioscience), anti-TNF-α-AF700 (clone Mab11) (BD Pharmingen) and anti-Perforin-PE (clone B-D48) (BioLegend). Finally, the PBMCs were fixed with a 4% paraformaldehyde solution (Sigma-Aldrich).

T cell specific response was evaluated as the frequency of cells that express intracellular cytokines (IFN-γ, IL-2, and TNF-α) or cytotoxicity markers after stimulation with overlapping Spike protein peptides, minus the levels of this response under unstimulated conditions (background subtraction). To classify an individual as a responder, the percentage of IFN-γ, IL-2, or TNF-α-secreting memory CD8^+^ and CD4^+^ T-cells must be two times higher than the percentage of unstimulated cells. The gating strategy is shown in **Supplementary Figure 3**.

Polyfunctionality was defined as the capacity of cells to simultaneously express intracellular cytokines, the degranulation marker CD107a, or the cytolytic protein perforin (PRF). Polyfunctionality pie charts were constructed using Pestle version 2.0 and Spice version 6.1 (provided by M. Roederer, NIH, Bethesda, MD) and quantified with the polyfunctional index (Pindex) algorithm employing the 0.1.2 beta version of the FunkyCells Boolean Dataminer software provided by Martin Larson (INSERM U1135, Paris, France).

### Statistical analysis

Categorical variables are expressed as numbers and percentages, and quantitative variables as median (M) and interquartile range (IQR). The χ2 test was used to compare categorical variables, and Fisher’s Exact test when needed. Single comparisons of independent groups was carried out by the Mann-Whitney *U* test, and the Wilcoxon matched-pairs signed-rank test for related samples. The Friedman test was used to compare changes in continuous variables over time in each treatment group. The association between continuous variables was assessed with Spearman’s rank correlation coefficients (rho). Linear regression models were carried out to estimate the association between the magnitude of immune response (anti-S-RBD IgG and nAb titers) and possible confounders such as age, CD4^+^ nadir, CD4^+^ T cell count, CD4^+^/CD8^+^ ratio, months with undetectable viral load, presence of comorbidities and vaccine received after logarithmic transformation of quantitative variables. Being this study an exploratory analysis, formal sample size estimation *a priori* was not performed. The Statistical Package for the Social Sciences software (SPSS) v. 26.0 (IBM, Madrid, Spa in) was used for the statistical analysis. Graphs were generated with Prism 9 (GraphPad Software 2021).

## Results

### Study population

Between 16 April and 30 June 2021, 89 participants were enrolled, of whom 28 were excluded due to incomplete follow-up, 13 due to positive anti-N antibodies at T_0_, and two vaccinated with Janssen. Furthermore, five cases were excluded from the T_2_ analysis due to positive anti-N antibodies. Finally, 46 participants (IR, 22; DIR, 24) were analyzed. All participants were on ART and had persistent undetectable viremia for a median of 130 months (IQR, 70−182) with no significant differences in other basal variables between the groups, except for CD4^+^ T cell counts and CD4^+^/CD8^+^ ratios that were quite different (p <0.0001). Furthermore, DIR received the mRNA-1273 vaccine more frequently than IR (p <0.0001) (**Table 1**). Although both groups had several comorbidities, we only selected those that could affect the immune response to vaccines, discarding others, such as hypertension.

**Table 1:** Baseline demographic, clinical characteristics and vaccines received.

	IR (n= 22)	DIR (n= 24)	p
**Male sex**, n (%)	20 (90.9)	16 (66.7)	0.074
**Age**, years	48 (42–53)	56 (47–60)	0.060
Risk for HIV infection, n (%)			
MSM	13 (59.1)	7 (29.2)	0.220
HTX	5 (22.7)	8 (33.30)	
Previous iv drug use	3 (13.6)	6 (25.0)	
Others	1 (4.5)	3 (12.5)	
**CD4^+^ T cell count**, cells/μL	654 (512–849)	168 (106–208)	< 0.001
**CD4^+^/CD8^+^ ratio**	1.12 (0.85–1.50)	0.30 (0.15–0.53)	< 0.001
**Nadir CD4^+^ T cells/μl**	258 (138–301)	47 (27–84)	< 0.001
**Highest HIV-RNA**, log_10_ copies/ml	4.95 (2.25–5.74)	5.37 (4.58–5.73)	0.391
**Months on treatment**	151 (79–171)	146 (45–218)	0.956
**HIV-RNA <50 copies/ml**, months	129 (71–153)	141 (41–201)	0.749
**AIDS-defining illness**, n (%)	4 (18.2)	9 (37.5)	0.197
**Comorbidities**, n (%)			0.706
Diabetes mellitus	1 (4.0)	3 (13.6)	
Liver cirrhosis with portal hypertension	−	2 (8.0)	
Chronic portal cavernomatosis with portal hypertension	−	1 (4.0)	
Visceral leishmaniasis (on treatment > 6 moths)	−	1 (4.0)	
**Antiretroviral treatment**, n (%)			0.343
InSTI + 2 NRTI	5 (22.7)	9 (37.5)	
InSTI + 1 NRTI	6 (27.3)	8 (33.3)	
NNRTI + 1 NRTI	2 (9.1)	2 (8.3)	
bPI + 1 NRTI	9 (40.9)	2 (8.3)	
InSTI + bPI		2 (8.3)	
InstI + NNRTI		1 (4.2)	
**mRNA-1273/BNT162b2 (1^st^ - 2^nd^ dose)**, n (%)	4/18 (18.2/81.8%)	19/5 (79.2/20.8%)	< 0.001
**mRNA-1273/BNT162b2 (3^rd^ dose),** n (%)	1/9 (10/90%)	10/6 (62.5/37.5)	0.011

IR, immunological responders; DIR, discordant immune responders; MSM, men who have sex with men; HTX, heterosexual; InSTI, integrase strand transfer inhibitor; NRTI, nucleos(t)ide reverse transcriptase inhibitors; bPI, boosted protease inhibitor.

DIR showed the expected T cell phenotype with a lower percentage of naïve CD4^+^ cells, inversion of the naïve/memory ratio cells, higher activation (HLA-DR^+^CD38^+^), and PD-1 expression in all CD4^+^ memory subsets, and a higher percentage of Tregs. Regarding CD8^+^, DIR also showed a lower percentage of naïve cells, inversion of the naïve/memory ratio cells, and higher activation of memory T cells. Likewise, total Tfh was similar in IR and DIR, but the latter group was characterized by a lower percentage of Tfh2 cells and higher Tfh1-17 cells (**Supplementary Tables 1–3**).

### Serological response to mRNA SARS-CoV-2 vaccines

**Figure 1:**
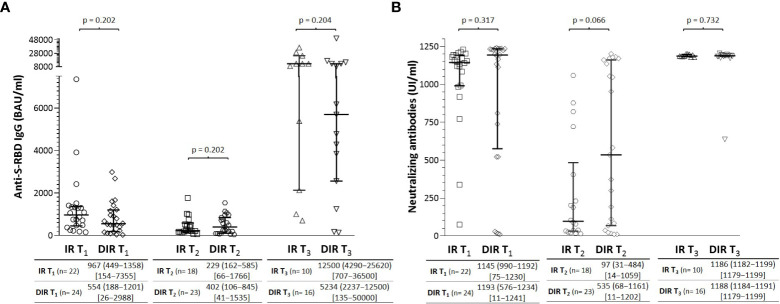
**(A)** Anti-S-RBD IgG and **(B)** neutralizing antibody titers one month (T1) and 5.5 months (T_2_) after two mRNA vaccine doses and one month after a third dose (T3) according to the group of participants (IR, immune responders. DIR, discordant immune responders).

At T_0_, T_1_, T_2_, and T_3_, plasma samples were tested for antibodies against the nucleoprotein antigen (N) to confirm a previous infection with SARS-CoV-2; participants with positive results were excluded from further analyses. A median of 33 days (30−36) (T_1_) after the two mRNA vaccine doses, all participants developed anti-S-RBD IgG antibodies without significant differences between IR and DIR participants (**Figure 1**). The putative protection threshold of 506 BAU/ml for an 80% vaccine efficacy against primary symptomatic COVID-19 (31) was achieved in 15/22 IR and 14/24 DIR (p = 0.552).

Neutralizing antibodies (nAb) against the receptor binding domain (RBD) of the SARS-CoV 2 spike protein S1 subunit were measured as a surrogate neutralization virus assay. At T_1_, 100% of subjects in the IR group developed nAb (**Figure 1**), with a median %IH of 95.9% (92.9−96.7). However, only 20/24 (83.3%) of DIR were positive for nAb (p = 0.065) with titers without significant differences with IR participants (p= 0.317) and %IH of 97.0% (95.4−97.4). Overall, anti-S-RBD IgG and nAbs titers were correlated with each other (ρ = 0.504, p <0.001).

After a median of 166 days (135−187) from the second dose (T_2_), the median anti-S-RBD IgG titers decrease in both IR and DIR (**Figure 1**), with a higher decrease in the IR group (-466 BAU/ml (-151 to -831) vs. -198 (221 to -852), although without reaching statistical significance (p= 0.227). The presumptive protection threshold of 506 BAU/ml was maintained more frequently in DIR 10/23 (43.5%) than in IR 5/18 (27.8%) (p = 0.346). Regarding the nAb titers, DIR maintained higher levels [535 IU/ml (68−1161)] with 18 participants (75%) showing neutralizing activity with a median %IH of 93.1% (66.4−96.3) compared to IR [97 IU/ml (31−484)], with 11/22 showing neutralizing activity with a %IH of 63.4% (47.1−89.1), p= 0.060].

**Figure 2:**
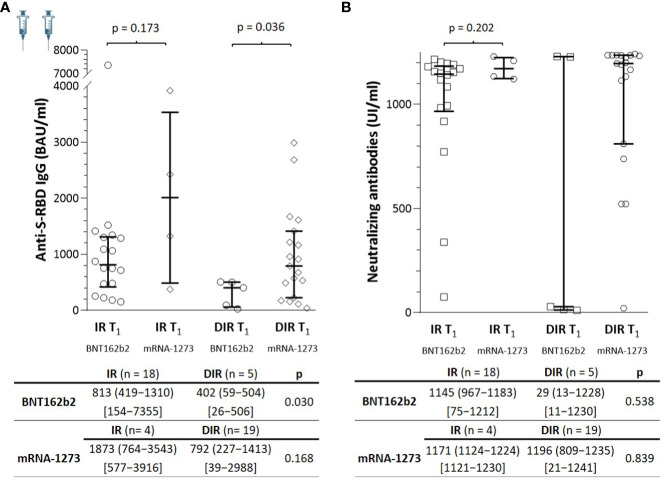
**(A)** Anti-S-RBD IgG and **(B)** neutralizing antibody titers one month (T1) after two mRNA vaccine doses according to the group of participants (IR, immune responders. DIR, discordant immune responders) and the vaccine received.

These surprising results led us to reanalyze them according to the vaccine received. At T_1_, the median anti-S-RBD IgG titers achieved with the mRNA-1273 were higher than those with the BNT162b2 vaccine in both IR [median 1873 BAU/ml (n = 4) vs. 813 (n = 18), p = 0.173] and DIR [792 BAU/ml (n = 19) vs. 402 (n = 5), p= 0.036] (**Figure 2**). Regarding nAb, in IR, titers were similar regardless of the vaccine administered [median, 1145 IU/ml vs. 1171, (p = 0.227)]. However, among DIR, the median nAb titers achieved with the mRNA-1273 vaccine were much higher (1196 IU/ml) than those achieved with BNT162b2 (29 IU/ml), although without reaching statistical significance (p = 0.110) due to the small sample size in the last group (n = 5) (**Figure 2**).

**Figure 3:**
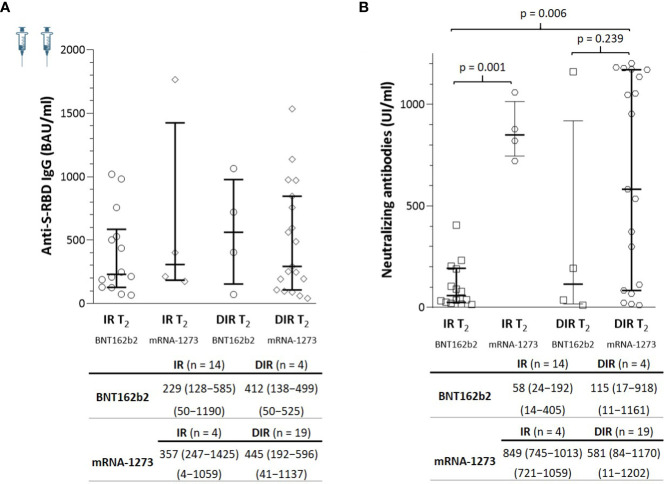
**(A)** Anti-S-RBD IgG and **(B)** neutralizing antibody titers a median of 5.5 months (T2) after two mRNA vaccine doses according to the group of participants (IR, immune responders. DIR, discordant immune responders) and the vaccine received.

At T_2_, the anti-S-RBD IgG titers were similar in IR and DIR, regardless of the vaccine administered. By contrast, the nAb titers were higher in those participants vaccinated with the mRNA-1273 compared to the BNT162b2 vaccine in both IR and DIR, reaching statistical significance only in the IR group (p = 0.001), but not in DIR (BNT162b2, median 115 UI/ml vs. mRNA-1273, 581; p = 0.239) probably due to the sample size of DIR (n = 4) vaccinatted with BNT162b2 (**Figure 3**).

Overall, bivariate analyses only showed associations between anti-S-RBD IgG titers at T_1_ with the basal CD4^+^ T cell count (ρ= 0.349, p= 0.017). To assess the influence of other variables on the results, we performed a multiple linear regression analysis in which the basal CD4^+^ T cell count, the vaccine received, and female gender were the variables related to the levels of anti-S-RBD at T_1_. Neither the percentages of naive B cells, SARS-CoV-2-specific memory B cells nor Tfh or Tregs influenced the response to vaccines. By contrast, the variables associated with the nAb titers were the basal CD4^+^ T cell count, the vaccine received, and the percentage of SARS-CoV-2-specific memory B cells. At T_2_, only the vaccine received, and the percentage of SARS-CoV-2-specific memory B cells influenced nAb titers (**Table 2**). Therefore, contrary to expectations, DIR had a similar response to IR, both anti-S-RBD IgG and neutralizing antibodies, with a better response in those participants vaccinated with mRNA-1273.

**Table 2:** Influence of different variables on the anti-S-RBD IgG and neutralizing antibodies (NAb) titers one month (T1) and 5.5 months (T2) after two doses of the BNT162b2 or mRNA-1273 vaccines.

Anti-S-RBD IgG (T_1_) (BAU/ml)	Beta	Sig.	R^2^	NAb (T_1_) (IU/ml)	Beta	Sig.	R^2^
**Gender** (female)	0.269	0.015	0.657		0.079	0.194	0.889
**Nadir CD4^+^ T cells/μl**	-0.501	0.023			-0.971	0.337	
**Basal CD4^+^ T cell count**	0.981	<0.001			0.565	<0.001	
**Vaccine** (mRNA-1273)	0.246	0.028			0.232	0.007	
**% SARS-CoV-2-specific** **memory-B cells**	-0.007	0.968			0.385	<0.001	
Anti-S-RBD IgG (T_2_) (BAU/ml)	Beta	Sig.	R^2^	NAb (T_2_) (IU/ml)	Beta	Sig.	R^2^
**Gender** (female)	0.116	0.299	0.662		0.129	0.161	0.772
**Nadir CD4^+^ T cells/μl**	0.118	0.606			0.047	0.963	
**Basal CD4^+^/CD8^+^ ratio**	0.173	0.723			-0.020	0.918	
**Vaccine** (mRNA-1273)	0.429	0.671			0.371	0.004	
**% SARS-CoV-2-specific** **memory-B cells**	0.516	0.003			0.507	0.001	

**Figure 4:**
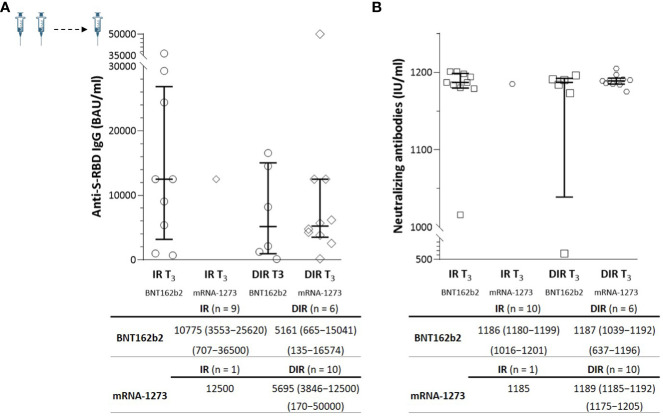
**(A)** Anti-S-RBD IgG and **(B)** neutralizing antibody titers one month (T3) after the third mRNA vaccine dose according to the group of participants (IR, immune responders. DIR, discordant immune responders) and the vaccine received.

In a subgroup of 10 IR (M CD4^+^ of 666/µl) and 16 DIR (M CD4^+^ of 171/µl) (**Supplementary Table 4**), a blood sample was available one month after a third additional dose (T_3_) administered a median of 204 days (175–212) after the second dose. Nine out of ten IR received the BNT162b2 vaccine, and ten out of 16 DIR received the mRNA-1273 vaccine. The third dose strongly boosted anti-S-RBD and nAb levels by a median of 20- and 6-fold, respectively, achieving titers without statistically significant differences between the groups or the vaccine received (**Figure 4**).

### Specific SARS-CoV-2 memory B cells

In addition to characterizing the longitudinal evolution of anti-S-RBD IgG and nAb, we analyzed B cells and SARS-CoV-2-specific B memory cells (**Table 3**). Overall, there was a linear correlation between the frequencies of naïve B cells before vaccination and the anti-S-RBD titers at T_1_ (ρ = 0.347, p = 0.015), which was more robust in IR (ρ = 0.917, p <0.001), but not for nAb.

**Table 3:** B-cell phenotype in immune responders (IR) and discordant immune responders (DIR).

	T_0_		T_1_		T_2_			
	IR (n= 9)	DIR (n= 22)	IR (n= 22)	DIR (n= 24)	IR (n= 18)	DIR (n= 23)	p (IR)	p (DIR)
**% B cells**	11.5 (8.6-17.8)	14.1 (9.6-19.6)	8.4 (4.9-14.0)	11.6 (8.0-15.0)	10.5 (6.4-15.5)	11.0 (7.1-14.1)	0.011	<0.001
**% Naïve B cells**	26.0 (21.5-41.4)	32.3 (22.8-37.8)	37.7 (26.2-42.3)	44.3 (27.1-51.7)	38.1 (27.1-41.5)	42.8 (34.6-52.1)	0.009	<0.001
**% Memory B cells**	18.3 (14.2-28.4)	14.7 (6.8-23.5)	20.0 (11.1-37.2)	15.6 (6.1-27.2)	18.8 (11.2-27.4)	15.2 (7.4-17.3)	0.513	0.073
**% ASC B cells**	0.2 (0.1-0.9)	0.3 (0.1-0.3)	0.2 (0.1-0.8)	0.3 (0.2-0.5)	0.1 (0.1-0.2)	0.3 (0.1-0.5)	0.368	0.580
**% SARS-CoV-2-specific memory B cells**	—	—	0.3 (0.2-0.3)	0.3 (0.2-0.7)	0.4 (0.3-0.6)	0.5 (0.4-1.1)	<0.001	0.144
**% SARS-CoV-2-specific memory B cells IgM^+^**	—	—	42.2 (24.2-50.0)	29.3 (12.2-48.5)	18.4 (9.0-38.6)	17.1 (7.1-27.6)	0.007	0.019
**%** S**ARS-CoV-2-specific memory B cells IgM^−^**	—	—	56.2 (50.0-75.8)	61.4 (25.7-80.0)	81.6 (61.4-91.0)	83.3 (69.0-92.9)	0.005	0.003

ASC, antibody-secreting cells.

**Figure 5:**
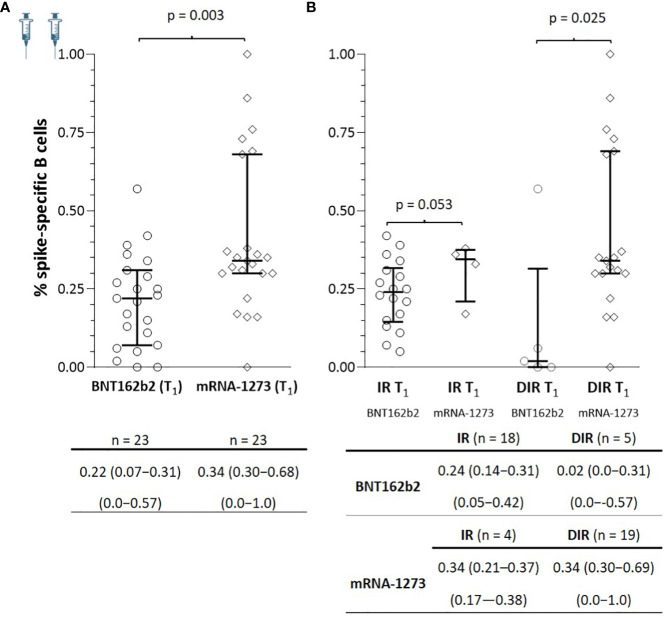
**(A)** Specific SARS-CoV-2 memory B cells one month after the two mRNA vaccine doses (T_1_) according to the vaccine received and **(B)** according to the group of participants (IR, immune responders. DIR, discordant immune responders) and the vaccine received.

At T_1_, spike-specific B cells were detected in all IR and 21/24 DIR (p = 0.235) with positive correlations between specific B memory cells and anti-S-RBD (ρ = 0.363, p = 0.010) and nAb (ρ = 0.557, p <0.001). As observed for antibodies, patients vaccinated with mRNA-1273 showed a higher percentage of specific memory B cells [0.34 (0.30−0.68)] than those vaccinated with BNT162b2 [0.22 (0.07−0.31], (p = 0.003). These differences were also notable regardless of the participant’s group, with a higher percentage of specific memory B cells in both IR and DIR vaccinated with mRNA1273 than those vaccinated with BNT162b2 (**Figure 5**). In this case, there were no relationships between the percentage of specific memory B cells and the CD4^+^ nadir, nor the basal CD4^+^ or CD4^+^/CD8^+^ ratio.

**Figure 6:**
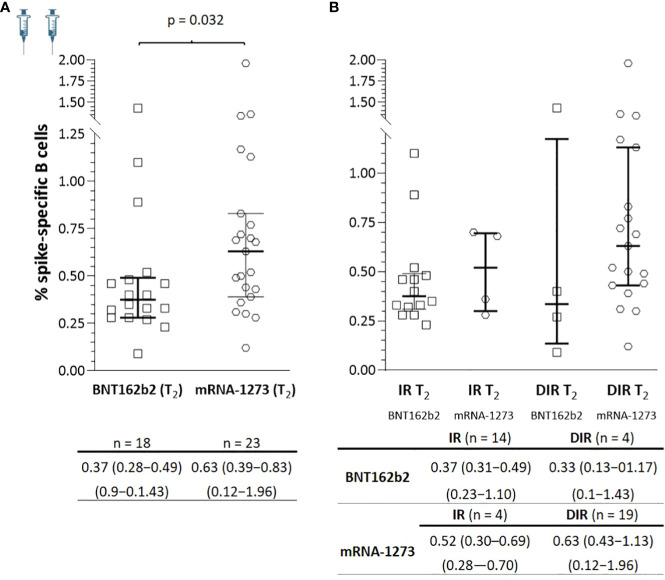
**(A)** Specific SARS-CoV-2 memory B cells a median of 5.5 months after the two mRNA vaccine doses (T_2_) according to the vaccine received and **(B)** according to the group of participants (IR, immune responders. DIR, discordant immune responders) and the vaccine received.

At T_2_, there was an increase in SARS-CoV-2-specific B memory cells in both IR and DIR (p <0.001), higher in DIR compared to IR (p = 0.080) and in those vaccinated with mRNA-1273 compared to BNT162b2 (p = 0.032) (**Figure 6**). Likewise, there were positive correlations between the percentage of SARS-CoV-2-specific B memory cells and anti-S-RBD (ρ = 0.363, p = 0.020) and the nAb titers (ρ = 0.697, p <0.001). Furthermore, specific B memory cells changed to a more mature pattern with higher IgM negative cells (p <0.001) (**Table 3**).

To complement the characterization of the humoral response in these subjects, we analyzed the different subpopulations of circulating T-follicular helper cells (cTfh) and their relationship with the humoral response. There were no correlations between the percentage of total cTfh or the different subsets with anti-S RBD IgG or nAb levels at T_0_ or T_1_ or with specific memory B cells, probably due to not having quantified of specific SARS-CoV-2 cTfh.

However, at T_2_, we observed a decrease in the Tfh1-17 subpopulation and an expansion of Tfh2 cells in DIR with positive (ρ = 0.490, p = 0.039) and negative correlations (ρ = -0.488, p 0.040) with anti-S-RBD IgG antibody levels, suggesting a role for this subset of Tfh in antibody production in these subjects (**Supplementary Table 3**).

### Plasma concentrations of IL-4 and IL-21

IL-4 and IL-21 are cytokines commonly secreted by T cells and Tfh cells after antigenic stimulation. Both have an essential role in the expansion, differentiation of B cells and production of memory B cells after exposure to an antigen or vaccination [26,27]. Therefore, we explored whether they could be used as markers of the efficacy of these vaccines, aware that their plasma concentrations do not necessarily reflect the concentrations and effects in lymphoid tissue. In general, IL-4 and IL-21 levels were higher in DIR than in IR at baseline, T_1_, and T_2_, reaching statistical significance only the differences in plasma concentrations of IL-21 at T_1_ (**Supplementary Figure 4**). There were no relationships between IL-4 and IL-21 with anti-S-RBD, nAb levels nor with the percentage of SARS-CoV-2-specific B cells at any time. Furthermore, there were no correlations between IL-4 and IL-21 with Tfh cells or their different subsets.

### SARS-CoV-2 specific T cell responses

We evaluated the SARS-CoV-2-specific memory CD4^+^ and CD8^+^ T cell responses in a subgroup of IR (n = 9; 7 vaccinated with BNT162b2) and DIR (n = 9; 7 vaccinated with mRNA-1273), whose demographic, immunological characteristics and the vaccine received are shown in **Supplementary Table 5**.

**Figure 7:**
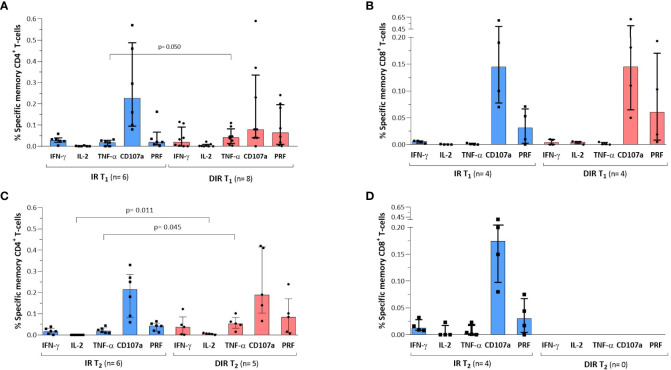
SARS-CoV-2 specific CD4^+^ and CD8 T^+^ cell responses after **(A, B)** one (T1) and **(C, D)** a median of 5.5 months (T2) after the two mRNA vaccine doses according to the group of participants (IR, immune 626 responders. DIR, discordant immune responders).

At T_1_, memory CD4^+^ T cells responded in five IR and seven DIR, mainly based on the expression of IFN-γ and TNF-α with a higher percentage of CD4^+^ expressing TNF-α in DIR [0.04% (0.01-0.08)] compared to IR [0.02% (0.00-0.03) (p = 0.050)] (**Figure 7A**). However, there was no relationship between the memory CD4^+^ T cell response and basal CD4^+^ counts, CD4^+^/CD8^+^ ratios, activation of total or memory CD4^+^, PD-1 expression, or percentage of Treg cells at baseline or T_1_.

When comparing the response as a function of the received vaccine, a higher percentage of cells expressed IFN-γ [0.04% (0.02−0.11)] and TNF-α [0.05% (0.03−0.10)] in participants vaccinated with mRNA-1273 compared to those immunized with BNT162b2 [0.02% (0.00−0.04) and 0.02% (0.00−0.03)], p = 0.132 and 0.026, respectively. Interestingly, participants who showed CD4^+^ T cell response had higher levels of anti-S-RBD [714 BAU/ml (503−1214)] and nAb [1194 IU/ml (1119−1224)] than those who did not show response [332 BAU/ml (145−867) and 1056 IU/ml (476−1172), p = 0.092 and 0.041, respectively], probably reflecting a less deteriorated immune system.

On the other hand, memory CD8^+^ T cells responded in only four participants in each group, five vaccinated with BNT162b2 and three with mRNA-1273, indicating that CD4^+^ T cells are the dominant population responding to SARS-CoV-2. Specific memory CD8^+^ cells showed low expression of these cytokines and predominant expression of the degranulation marker CD107a and the cytolytic protein perforin, without differences between vaccines (**Figure 7B**). There were no relationships between cytokine expression and the presence of one or more expression of exhaustion markers (PD-1, TIGIT, LAG-3, and TIM) in memory CD4^+^ or CD8^+^ T cells.

At T_2_, six IR and five of the eight DIR who showed specific memory CD4^+^ T cell responses at T_1_ also showed response. The percentage of memory CD4^+^ expressing the different cytokines was lower in both groups compared to T_1_, except for TNF-α, which was somewhat higher at T_2_. DIR showed higher expression of TNF-α [0.05% (0.03-0.08)] and IL-2 [0.01% (0.00-0.01)] than IR [0.02% (0.00-0.03) and 0.00 (0.00-0.00), (p = 0.045 and 0.011)], respectively **Figure 7C**). Regarding CD8^+^, we only observed response in four of the nine IR with a slight increase in the IFN-γ expression and a somewhat lower percentage of cells expressing CD107a and perforin compared to T_1_. In the four DIR who showed CD8^+^ response at T_1_, three vaccinated with BNT162b2 and one with mRNA-1273, specific CD8^+^ responses were lost (**Figure 7D**). Once again, there were no relationships between cytokine expression and the presence of one or more exhaustion markers.

**Figure 8:**
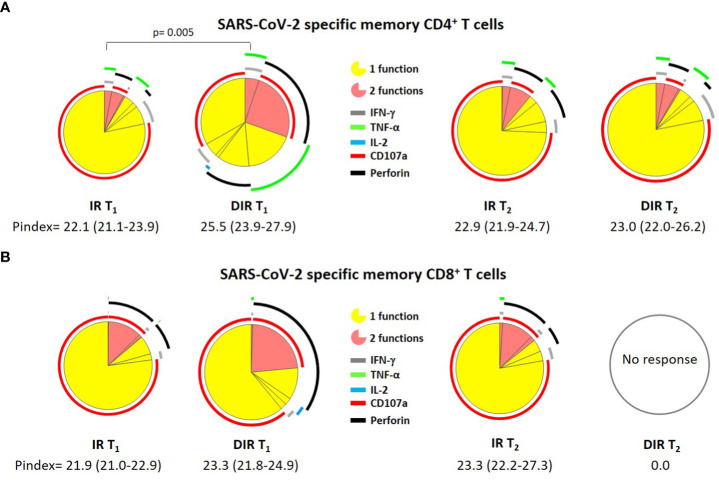
Pie charts representing **(A)** SARS-CoV-2 S–specific memory CD4^+^ and **(B)** CD8^+^ T cell polyfunctionality. Each sector represents the proportion of S-specific CD4^+^ T cells producing 1 (yellow) or 2 (red) functions. Arcs represent the type of function (IFN-γ, IL-2, TNF-α, CD107a, and perforin) expressed in each sector. IR, immune responders. DIR, discordant immune response. T_1_ and T_2_ a median of 33 and 166 days after the second ARN vaccine dose.

The next step was to investigate the quality of these responses using the polyfunctionality index and the cytokines combination analysis, defined as the ability of cells to produce multiple cytokines and degranulate simultaneously. In this sense, the specific memory CD4^+^ T cell P index was higher in DIR [25.5 (23.9-27.9)] than in IR [22.1 (21.1-23.9), (p = 0.005)], but similar for CD8^+^ T cells (**Figure 8**), without differences between vaccines.

At T_2_, the CD4^+^ polyfunctionality index was similar to T_1,_ with a P index [22.9 (21.9-24.7)] for IR and 23.0 (22.0-26.2) for DIR. Likewise, the memory CD8^+^ P index resembles T_1_ in IR [23.3 (22.2-27.3], while we did not observe any response in DIR (**Figure 8**).

## Discussion

Severe clinical outcomes occurred commonly in PLWH with COVID-19, especially with low CD4^+^ T-cell counts and in older subjects (32, 33). Accordingly, these PLWH received priority access to SARS-CoV-2 vaccines in many vaccine programs, including the Spanish one. With mRNA vaccines, it has been observed that PLWH with CD4^+^ T cell count >500/µl and undetectable viremia have a serological response comparable to or slightly lower than healthy controls (12, 18). Among the studies that have included more PLWH with <200 CD4^+^ T cells are those by Corma-Gomez et al. (23) and Hassold et al. (20). In the first, 28 PLWH received an mRNA vaccine, 23 of them with an undetectable viral load, and samples taken between 4 and 8 weeks. In the second, 16 received an RNA vaccine, but only 8 had a viral load <50 copies/ml; the samples were taken a median of 73 days (29–97) after the second dose. Both studies showed lower seroconversion rates and anti-RBD and nAb titers in the CD4^+^ <200 groups.

It is noteworthy the study by Antinori et al. (34), which included 32 PWL with <200 CD4^+^/µl, 69% with undetectable viremia. In addition to serological studies, they incubated whole blood with a pool of SARS-CoV-2 spike protein peptides and measured IFN-γ and IL-2 production in the supernatant by ELISA. They concluded that CD4^+^ T cell <200/µl significantly predicted lower anti-S RBD-binding IgG and nAb neutralizing titers and IFN-γ response one month after the second vaccine dose. A more recent study by Benet et al. (35) included 58 PLWH with <200 CD4^+^ and 36 with CD4^+^ counts >500/µl, mostly vaccinated with the mRNA-1273 vaccine. One month after the second vaccine dose, one-third of PLWH with CD4^+^ <200/µl showed low anti-S/anti-RBD IgG levels, reduced *in vitro* neutralization activity against SARS-CoV-2, and no vaccine-induced T cell response measured by an IFN-γ ELISPOT kit.

Regarding PLWH with a discordant immune response (DIR), the response to SARS-CoV-2 RNA vaccines has been specifically addressed in a small series of 10 subjects vaccinated with two doses of BNT162b2 (30). In 5 of them, no spike-specific IgG titers or neutralizing activity was observed. The other five generated strong neutralizing antibody responses with % IH values close to 100%, similar to healthy controls, with no clear correlation between vaccine response and CD4 T cell counts.

There is insufficient information directly examining the serological response, the specific B cells, and the response of CD4^+^ and CD8^+^ T cells to SARS-CoV-2 in the same PLWH, particularly in DIR. Therefore, we comprehensively analyzed the three branches of the adaptive immune response against SARS-CoV-2 in these subjects.

In our series, one month after the second RNA vaccine, all participants developed anti-S-RBD IgG antibodies with levels in IR higher than those in DIR. The putative protection threshold of 506 BAU/ml was achieved in 68% of IR and 63% of DIR. By contrast, the nAb titers were similar in both groups. After a median of 5.5 months, the levels of both types of antibodies decrease in both groups. However, DIR maintained higher titers of anti-S-RBD IgG (43.5% >506 BAU/ml vs. 27.8% in IR), higher nAb levels, a greater percentage of participants showing neutralizing activity, and a trend towards a higher percentage of specific B cells.

Regarding SARS-CoV-2 specific T cell responses, CD4^+^ T cells were the predominant population that showed response, and, once again, DIR exhibited a higher rate of response and expression of TNF-α. At T_2_, the number of participants showing a specific response remained among IR and decreased from eight to five among DIR, but with a somewhat higher expression of TNF-α and IL-2. At T_1_, the CD8^+^ T cell response was observed only in four participants (44%) in each group, with the predominant expression of CD107a and perforin. However, at T_2_, the CD8^+^ response was maintained in the four IRs but lost in DIR.

These unexpected results led us to analyze the response according to the vaccine received, realizing that the vaccine administered had an outstanding influence on the humoral response and the specific-memory B cells generated, with better results in those vaccinated with the mRNA-1273. To our knowledge, no randomized trials have compared the BNT162b2 and mRNA-1273 vaccines head-to-head. However, there are references to a better performance of mRNA-1273 compared to the BNT162b2 vaccine not only in PLWH (18, 36) but also in solid organ transplant recipients (37) and healthy donors (38, 39). Furthermore, the mRNA-1273 vaccine appears to result in significantly lower rates of SARS-CoV-2 infection and related hospitalization (40). These results could be due to their higher mRNA antigenic load (100 µg for mRNA-1273 and 30 µg for BNT162b2), the nucleoside sequence, or the different lipid formulation (41, 42).

Regarding plasma levels of IL-4 and IL-21, despite the fundamental role they play in the differentiation of B lymphocytes and the production of memory B lymphocytes, unfortunately, they have not been helpful as markers of the efficacy of these vaccines.

In Spain, the regulatory authorities indicated a third additional dose to maintain adequate protection for SARS-CoV-2 and the VoCs, especially for susceptible subpopulations. As previously reported (43), this additional dose considerably increased anti-S RBD Ig G and nAb levels, reaching values higher than those observed one month after the second dose. Unfortunately, we did not have available samples to analyze the specific B and T responses.

The current study has some limitations. First, we did not include a control group of healthy individuals, as the study was designed to specifically evaluate the DIR group, which is poorly represented in the literature, and multiple studies have compared PLWH with elevated CD4^+^ T cell counts and healthy controls. Furthermore, many comparisons do not reach statistical significance due to the small sample size of some subgroups because the study was not designed to compare vaccines, which were administered at the discretion of the health authorities. Finally, we have not considered the role of active smoking on response to these vaccines, given the growing body of evidence showing a negative impact of this habit on vaccine response (44).

However, to our knowledge, the present study is the first to show a broad picture of humoral, specific B and T cell responses in this group of PLWH, with an extended follow-up, until the third dose of vaccine, which, given the data presented, appears entirely justified.

In conclusion, in both groups of participants, IR and DIR, the anti-S-RBD IgG titers achieved at T1, the nAb titers at T1 and T2, the percentage of specific memory B cells, and the SARS-CoV-2-specific CD4+ T cell responses were higher among those vaccinated with mRNA-1273 compared to BNT162b2, although in some cases these differences did not reach statistical significance due to the small sample size in some groups. Our data suggest that PLWH with undetectable viremia for a long time and a discordant immune response can generate an immune response similar to those with higher CD4^+^ T cell counts, provided that they are vaccinated with mRNA-1273 rather than with others less immunogenic. Even so, these individuals would benefit from monitoring vaccine responsiveness and prioritizing additional booster vaccinations against SARS-CoV-2

## Data availability statement

The original contributions presented in the study are included in the article/**Supplementary Material**. Further inquiries can be directed to the corresponding author.

## Ethics statement

The studies involving human participants were reviewed and approved by Ethics Committee for Clinical Research of the Virgen Macarena and Virgen del Rocío University Hospitals (CEI VM-VR_04/2021_N) and the National Health Authority. The patients/participants provided their written informed consent to participate in this study.

## Author contributions

LL-C: Conception and design, provision of study patients, data analysis and interpretation and manuscript writing. AS-A, MT-R, and AS-G: Collection of data, performed the laboratory determinations, data analysis and interpretation, manuscript writing. SL-F: Administrative support, collection and/or assembly of the data. EM-M: Collection and/or assembly of data, data analysis and interpretation. MP-S, CL, and MM-T: Collection of data and performed the laboratory determinations. CR and NE: Provision of study patients. AG-V: Conception and design, data analysis and interpretation and manuscript writing. All authors contributed to the article and approved the submitted version.
