# Supplementary material for: Humoral and cellular immunity to SARS-COV-2 after vaccination with mRNA vaccines in PLWH with discordant immune response. Influence of the vaccine administered
Source: Front Immunol. 2023 Mar 15;14:1129753. doi: 10.3389/fimmu.2023.1129753 (PMC10050444; doi:10.3389/fimmu.2023.1129753)
Supplement: Supplementary file 1 [file DataSheet_1.docx]

**Supplementary material**

**
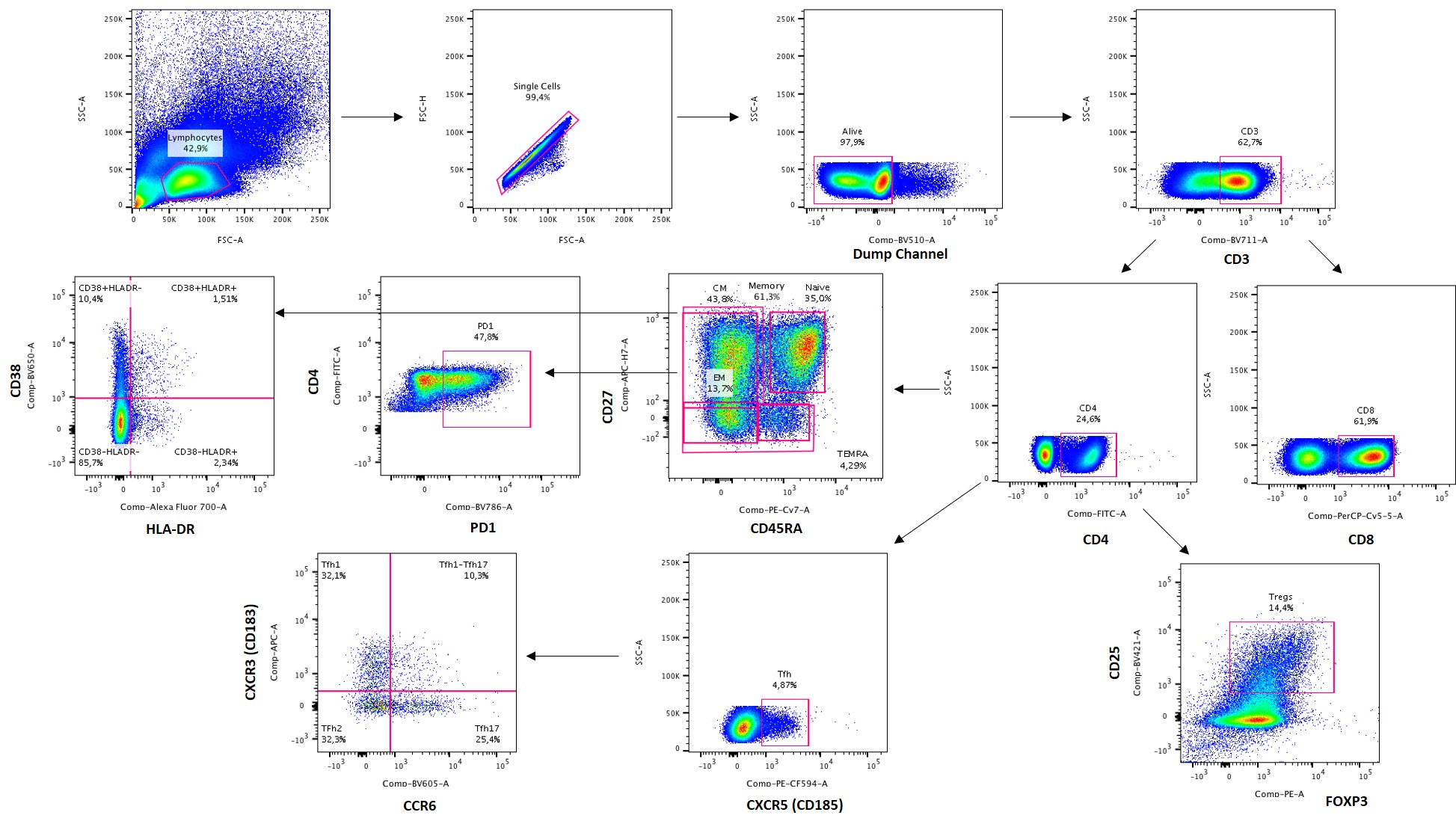
**

**Supplementary Figure 1.** T-cell phenotype gating strategy. Living lymphocytes were gated using the FSC-A/SSC-A axis, followed by a viability marker. Then, CD8+ (CD3+ CD8+) and CD4+ (CD3+ CD4+) T cells were selected, and T cell subsets were identified based on the expression of CD45RA and CD27: naive (CD45RA+ CD27-), central memory (CM) (CD45RA- CD27+), effector memory (EM) (CD45RA- CD27-), and terminally differentiated effector memory (TEMRA) (CD45RA+ CD27+) cells. Total memory cells (memory) correspond to the sum of CM, EM, and TEMRA T cells. Activation (HLA-DR, CD38) and exhaustion (PD1) markers were analyzed for each cell subset. In addition, Treg cells were identified by CD4, CD25, and Foxp3 expression, and Tfh cells were gated on CD4, CXCR5, CXCR3, and CCR6 expression.

**
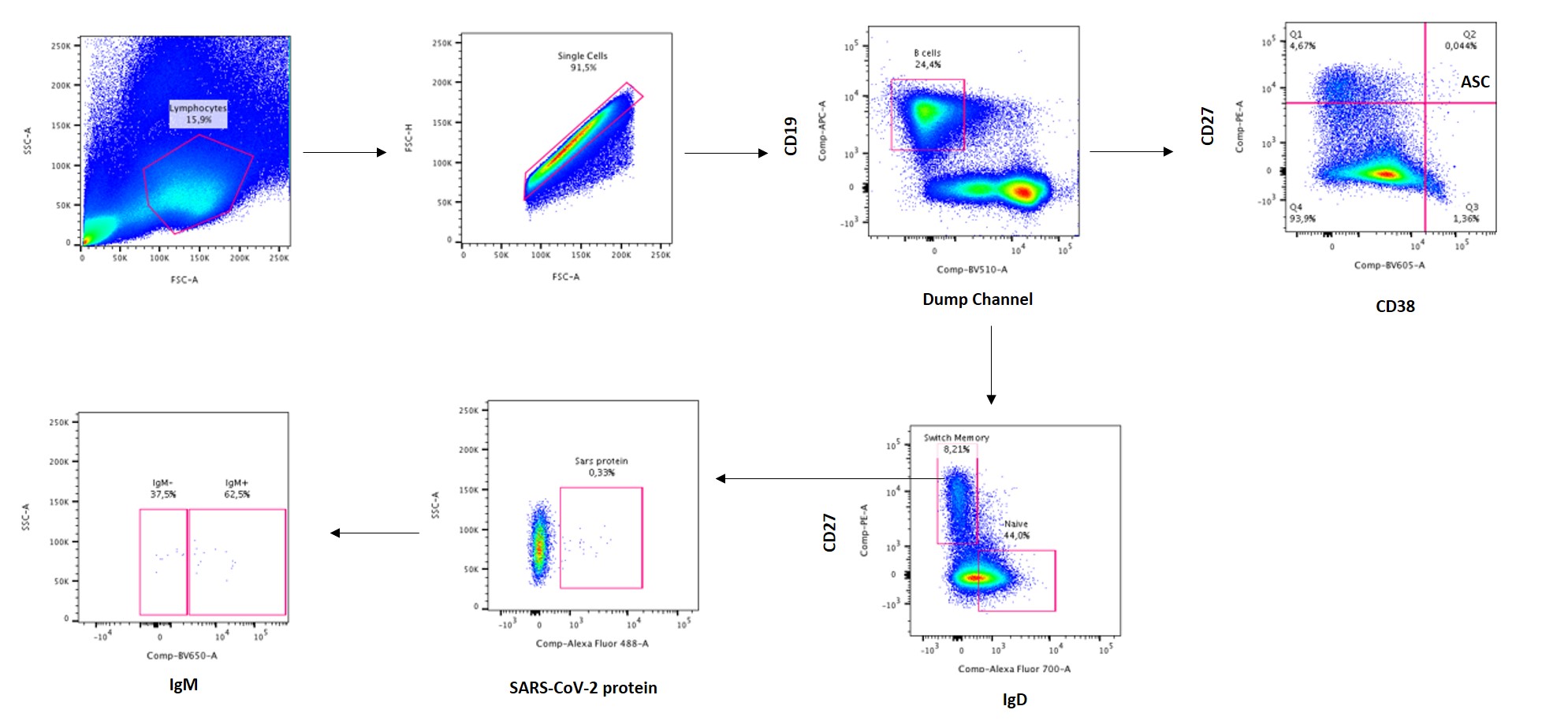
**

**Supplementary Figure 2.** Supplementary Figure 1. B-cell phenotype gating strategy. Cells were first visualized on FSC-A vs SSC-A to select lymphocyte population, and cells negative for dump channel (viability, CD14, CD3, and CD56) and positive for CD19 were gated. Later, B cell subsets [(naive, memory, and antibody-secreting cells (ASC)] were identified based on the expression of IgD, CD27, and CD38, and of the memory cells, those SARS-CoV-2 protein-positive cells were selected. Finally, SARS-CoV-2 protein-positive cells were separated in the function of IgM expression.

**
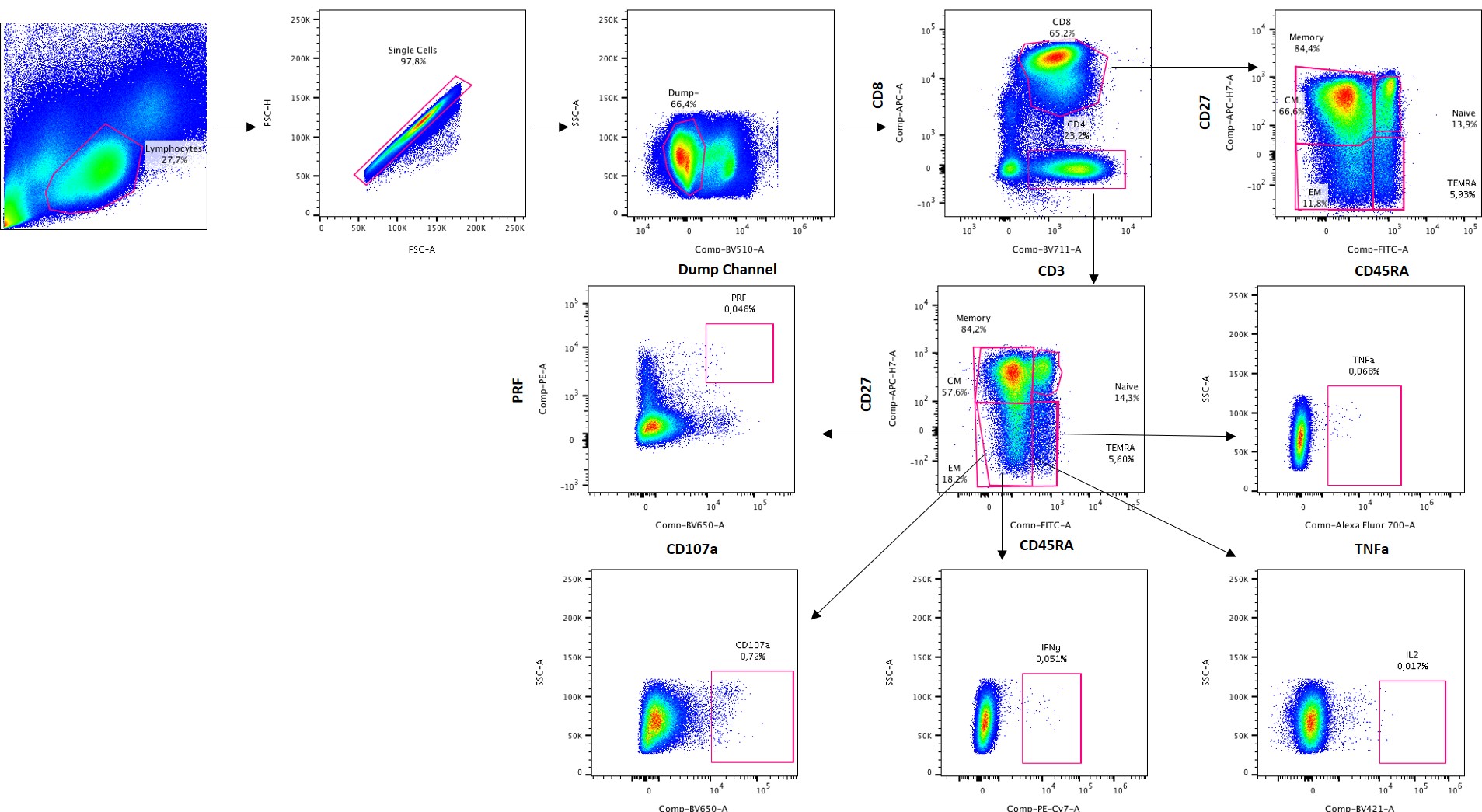
**

**Supplementary Figure 3.** T-cell response gating strategy. Cells were first visualized on FSC-A vs SSC-A to select lymphocyte population, and cells negative for dump channel (viability, CD14, CD19, and CD56) were gated. Then, CD8+ (CD3+ CD8+) and CD4+ (CD3+ CD8-) T cells were selected, and T cell subsets were identified based on the expression of CD45RA and CD27: naive (CD45RA+ CD27-), central memory (CM) (CD45RA- CD27+), effector memory (EM) (CD45RA- CD27-), and terminally differentiated effector memory (TEMRA) (CD45RA+ CD27+) cells. Total memory cells (Memory) correspond to the sum of CM, EM, and TEMRA T cells. For each cell subset, intracellular cytokine production (IFN-γ, IL-2, and TNF-α) and cytotoxicity markers (CD107a and perforin) were analyzed.

|  | **IR** (n= 22) | **DIR** (n= 24) | **p** |
| --- | --- | --- | --- |
| **(%) CD4^+^** **T cell** | 46.4 (41.0-53.5) | 19.4 (11.7-29.0) | <0.001 |
| **(%) CD4^+^ HLA-DR^+^CD38^+^** | 0.3 (0.2-0.4) | 1.0 (0.5-1.6) | <0.001 |
| **(%) CD4^+^ PD1^+^** | 11.4 (7.6-17.4) | 25.7 (23.0-35.8) | <0.001 |
| **(%) CD4^+^_N_** | 26.2 (13.8-36.8) | 15.9 (6.8-24.6) | 0.030 |
| **(%) CD4^+^_M_** | 70.4 (59.7-84.3) | 82.0 (72.7-90.5) | 0.020 |
| **(%) CD4^+^_M_ HLA-DR^+^CD38^+^** | 0.3 (0.2-0.5) | 1.1 (0.6-2.0) | <0.001 |
| **(%) CD4^+^_M_ PD1^+^** | 18.3 (10.2-24.3) | 34.4 (26.4-45.0) | <0.001 |
| **(%) CD4^+^_CM_** | 48.4 (42.3-58.4) | 55.9 (48.5-64.1) | 0.042 |
| **(%) CD4^+^_CM_ HLA-DR^+^CD38^+^** | 0.3 (0.2-0.5) | 0.8 (0.5-1.4) | <0.001 |
| **(%) CD4^+^_CM_ PD1^+^** | 14.9 (12.1-23.0) | 34.7 (27.3-41.6) | <0.001 |
| **(%) CD4^+^_EM_** | 17.9 (10.0-23.5) | 19.9 (12.9-32.1) | 0.361 |
| **(%) CD4^+^_EM_ HLA-DR^+^CD38^+^** | 0.8 (0.5-1.3) | 1.7 (0.8-3.6) | 0.003 |
| **(%) CD4^+^_EM_ PD1^+^** | 30.3 (18.9-41.1) | 42.2 (27.8-55.1) | 0.018 |
| **(%) CD4^+^_TEMRA_** | 0.9 (0.5-3.4) | 0.6 (0.3-2.5) | 0.084 |
| **(%) CD4^+^_TEMRA_** **HLA-DR^+^CD38^+^** | 1.3 (0.8-2.9) | 10.8 (3.6-26.3) | <0.001 |
| **(%) CD4^+^_TEMRA_ PD1^+^** | 5.7 (1.8-15.9) | 13.2 (3.9-22.6) | 0.132 |
| **(%) Regulatory T cells** | 5.1 (4.3-7.4) | 7.6 (5.4-11.7) | 0.012 |
| **(%) T follicular helper cells** | 8.5 (5.2-11.2) | 8.3 (4.0-11.7) | 0.904 |

**Supplementary Table 1**. CD4^+^ and CD8^+^ T-cell phenotypes at T_1_ in immune responders (IR) and discordant immune responders (DIR). N, naïve, M, memory. CM, central memory. EF, effector memory. TEMRA, terminally differentiated effector memory.

|  | **IR** (n= 22) | **DIR** (n= 24) | **p** |
| --- | --- | --- | --- |
| **(%) CD8^+^** **T cell** | 28.6 (22.7-36.9) | 43.2 (31.6-67.8) | 0.001 |
| **(%) CD8^+^ HLA-DR^+^CD38^+^** | 0.4 (0.3-0.8) | 1.0 (0.5-1.4) | 0.017 |
| **(%) CD8^+^ PD1^+^** | 17.3 (12.0-24.0) | 19.5 (16.0-27.1) | 0.312 |
| **(%) CD8^+^_N_** | 15.6 (9.2-26.4) | 7.3 (4.4-21.2) | 0.017 |
| **(%) CD8^+^_M_** | 74.4 (61.8-83.0) | 83.8 (65.6-92.0) | 0.050 |
| **(%) CD8^+^_M_ HLA-DR^+^CD38^+^** | 1.0 (0.7-1.5) | 1.6 (0.9-2.7) | 0.090 |
| **(%) CD8^+^_M_ PD1^+^** | 20.0 (15.7-37.1) | 23.4 (19.6-43.0) | 0.435 |
| **(%) CD8^+^_CM_** | 28.2 (22.5-36.1) | 31.1 (23.0-37.4) | 0.750 |
| **(%) CD8^+^_CM_ HLA-DR^+^CD38^+^** | 1.3 (0.7-2.0) | 2.0 (1.2-3.1) | 0.065 |
| **(%) CD8^+^_CM_ PD1^+^** | 32.1 (21.9-46.0) | 38.2 (19.6-43.0) | 0.895 |
| **(%) CD8^+^_EM_** | 27.4 (15.9-34.4) | 25.3 (19.7-35.5) | 0.553 |
| **(%) CD8^+^_EM_ HLA-DR^+^CD38^+^** | 1.1 (0.5-2.3) | 1.5 (0.9-2.8) | 0.166 |
| **(%) CD8^+^_EM_ PD1^+^** | 26.1 (17.2-38.8) | 24.3 (16.8-35.1) | 0.800 |
| **(%) CD8^+^_TEMRA_** | 12.5 (6.5-22.0) | 20.3 (8.8-24.8) | 0.187 |
| **(%) CD8^+^_TEMRA_** **HLA-DR^+^CD38^+^** | 0.6 (0.5-1.1) | 1.3 (0.6-2.1) | 0.048 |
| **(%) CD8^+^_TEMRA_ PD1^+^** | 7.3 (2.2-11.9) | 7.4 (3.4-15.3) | 0.429 |

**Supplementary Table 2**. CD4^+^ and CD8^+^ T-cell phenotype at T_1_ in immune responders (IR) and discordant immune responders (DIR). N, naïve, M, memory. CM, central memory. EF, effector memory. TEMRA, terminally differentiated effector memory.

|  | **IR T_0_**  **(n= 9)** | **DIR T_0_**  **(n= 22)** | **p (T_0_)** | **IR T_1_**  **(n= 22)** | **DIR T_1_**  **(n= 24)** | **p (T_1_)** | **IR T_2_**  **(n=18)** | **DIR T_2_**  **(n= 23)** | **p (T_2_)** | **p IR**  **(T_0-1-2)_)** | **p DIR**  **(T_0-1-2)_)** |
| --- | --- | --- | --- | --- | --- | --- | --- | --- | --- | --- | --- |
| **% Total cTfh** | 9.1  (4.5–11.2) | 9.0  (6.8–14.8) | 0.408 | 8,5  (5.1–11.7) | 8.3  (4.0–11.7) | 0.904 | 4.4  (1.7–6.0) | 4.8  (3.1–8.3) | 0.242 | 0.042 | 0.006 |
| **% Tfh1** | 22.3  (15.5–23.6) | 25.9  (15.5–34.5) | 0.240 | 24.2  (17.7–26.9) | 21.2  (10.8–33.1) | 0.758 | 18.3  (16.0–21.0) | 20.4  (15.1–36.1) | 0.318 | 0.513 | 0.717 |
| **% Tfh2** | 44.6  (40.7–53.8) | 33.1  (27.5–47.0) | 0.048 | 44.7  (40.3–55.4) | 33.7  (28.2–51.6) | 0.013 | 61.9  (49.5–72.3) | 44.7  (37.6–55.2) | 0.007 | 0.016 | 0.007 |
| **% Tfh17** | 28.5  (23.5–33.5) | 27.0  (16.3–37.0) | 0.896 | 26.3  (18.5–27.2) | 26.3  (19.3–34.9) | 0.166 | 13.1  (8.3–24.4) | 24.5  (11.3–30.1) | 0.128 | 0.115 | 0.129 |
| **% Tfh1–17** | 5.7  (3.3–7.1) | 9.3  (5.0–11.4) | 0.082 | 6.1  (3.7–8.1) | 9.1  (4.5–11.4) | 0.018 | 5.3  (3.2–8.2) | 5.4  (2.7–8.0) | 0.012 | 0.069 | <0.001 |

**Supplementary Table 3**. Percentage of total circulating T follicular helper cells (cTfh) in IR (immunological responders) and DIR (discordant immune response) at baseline (T_0_), one month (T_1_) and a median of 5.5 months (T_2_) after the two doses of mRNA vaccines. cTfh cells were classified based on the CXCR3 and CCR6 expression into four subsets: Tfh1 (CXCR3^+^CCR6^−^), Tfh2 (CXCR3^−^CCR6^−^), Tfh17 (CXCR3^−^CCR6^+^) and Tfh1-17 (CXCR3^+^CCR6^+^). Tfh1, Tfh2, Tfh17, Tfh1–17 expressed as percentage of total cTfh. Data expressed as median (IQR).

|  | **IR** (n= 10) | **DIR** (n= 16) | **p** |
| --- | --- | --- | --- |
| **Male sex**, n (%) | 9 (90) | 11 (68.8.8) | 0.352 |
| **Age**, years | 48 (43–54) | 58 (55–62) | 0.017 |
| **CD4^+^ T cell count**, cells/μL | 666 (511–809) | 171 (94–260) | < 0.001 |
| **CD4^+^/CD8^+^ ratio** | 1.04 (0.78–1.47) | 0.31 (0.23–0.47) | < 0.001 |
| **Nadir CD4^+^ T cells/μl** | 264 (150–284) | 47 (16–68) | < 0.001 |
| **HIV-RNA <50 copies/ml**, months | 137 (126–159) | 166 (106–214) | 0.421 |
| **Comorbidities**, n (%) |  |  | 0.562 |
| Diabetes mellitus | 2 (13.6) | 0 |  |
| **mRNA-1273/BNT162b2 (1^st^ - 2^nd^ dose)**, n (%) | 2/8 (20/80) | 12/4 (75/25) | 0.014 |
| **mRNA-1273/BNT162b2 (3rd dose)**, n (%) | 1/9 (10/90) | 10/6 (62.5/37.5) | 0.014 |

Supplementary table 4. Baseline demographic, clinical characteristics and vaccines received at T_3_. IR, immunological responders. DIR, immunological non-responders.


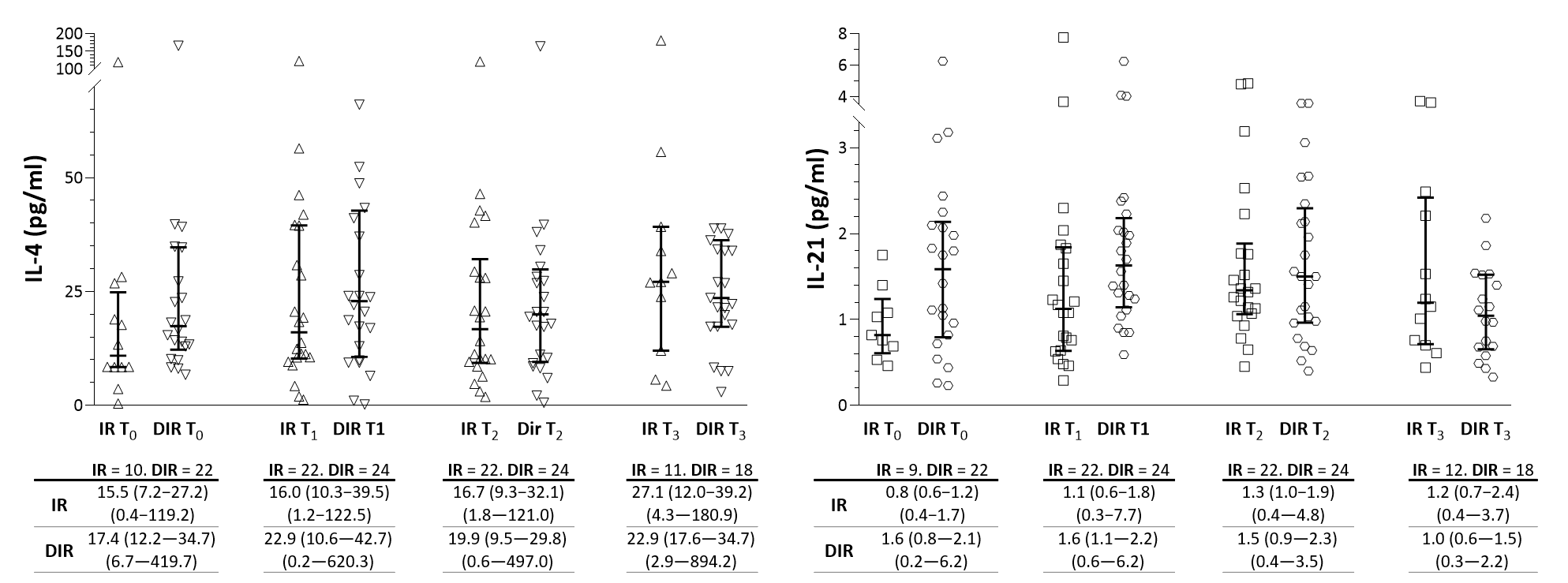


**Supplementary Figure 4**. IL-4 and IL-2 levels at baseline (T_0_), one month (T_1_) and a median of 5.5 months (T_2_) after the two doses of mRNA vaccines according to the group of participants (IR, immune responders. DIR, discordant immune responders).

|  | **IR** (n= 9) | **DIR** (n= 9) | **p** |
| --- | --- | --- | --- |
| **Male sex**, n (%) | 8 (88.9) | 7 (77.8) | 0.500 |
| **Age**, years | 50 (43–56) | 52 (44–57) | 0.605 |
| **CD4^+^ T cell count/μl** | 545 (475–928) | 162 (127–195) | < 0.001 |
| **CD4^+^/CD8^+^ ratio** | 1.10 (0.75–1.55) | 0.28 (0.10–0.35) | < 0.001 |
| **Nadir CD4^+^ T cells/μl** | 267 (195–445) | 40 (30–72) | 0.003 |
| **HIV-RNA <50 copies/ml**, months | 133 (95–221) | 141 (27–202) | 0.546 |
| **Comorbidities**, n (%) |  |  | 0.235 |
| Diabetes mellitus | 2 (13.6) | 0 |  |
| **mRNA-1273/BNT162b2 (1^st^ - 2^nd^ dose)**, n (%) | 2/7 (22.2/77.8%) | 7/2 (77.8/22.2%) | 0.028 |

Supplementary table 5. Baseline demographic, clinical characteristics and vaccines received in IR (immunological responders) and DIR (discordant immunologic responders) in whom the SARS-CoV-2 specific CD4^+^ and CD8^+^ T cell responses were analyzed.
